# Bazi Bushen alleviates skin senescence by orchestrating skin homeostasis in SAMP6 mice

**DOI:** 10.1111/jcmm.17833

**Published:** 2023-08-23

**Authors:** Zhe Xu, Boyang Gong, Zhaodong Li, Ying Wang, Zeyu Zhao, Lulu Xie, Yanfei Peng, Shuwu Zhao, Huifang Zhou, Yuhong Bian

**Affiliations:** ^1^ School of Integrative Medicine Tianjin University of Traditional Chinese Medicine Tianjin China; ^2^ School of Medicine Nankai University Tianjin China

**Keywords:** antioxidant, hemidesmosome component collagen XVII (COL17A1), senescence‐associated secretory phenotype (SASP), skin homeostasis, skin senescence, traditional Chinese medicine

## Abstract

Bazi Bushen, a Chinese‐patented drug with the function of relieving fatigue and delaying ageing, has been proven effective for extenuating skin senescence. To investigate the potential mechanism, senescence‐accelerated mouse prone 6 (SAMP6) was intragastrically administered with Bazi Bushen for 9 weeks to induce skin homeostasis. Skin homeostasis is important in mitigating skin senescence, and it is related to many factors such as oxidative stress, SASP, apoptosis, autophagy and stem cell. In our study, skin damage in SAMP6 mice was observed using HE, Masson and SA‐β‐gal staining. The content of hydroxyproline and the activities of SOD, MDA, GSH‐PX and T‐AOC in the skin were measured using commercial assay kits. The level of SASP factors (IL‐6, IL‐1β, TNF‐α, MMP2 and MMP9) in skin were measured using ELISA kits. The protein expressions of p16, p21, p53, Bax, Bcl‐2, Cleaved caspase‐3, LC3, p62, Beclin1, OCT4, SOX2 and NANOG were measured by western blotting. The expression of ITGA6 and COL17A1 was measured by immunofluorescence staining and western blotting. Our findings demonstrated that Bazi Bushen alleviated skin senescence by orchestrating skin homeostasis, reducing the level of oxidative stress and the expression of SASP, regulating the balance of apoptosis and autophagy and enhancing the protein expressions of ITGA6 and COL17A1 to improve skin structure in SAMP6 mice. This study indicated that Bazi Bushen could serve as a potential therapy for alleviating skin senescence.

## 1 INTRODUCTION

Ageing is a process defined as the time‐dependent gradual functional deterioration at the cellular and organismal levels. The improvement of health status and the extension of life expectancy are worth celebrating. Nevertheless, a longer life span in humans has led to a global burden of late‐life diseases.^1^ Ageing, which impairs sensory, motor and cognitive function, is the predominant risk factor for many chronic diseases.^2^ Skin ageing is one of the important manifestations of ageing, accompanied by cumulative changes in skin structure, function and appearance such as increased wrinkles, elastosis and telangiectasia.^3^ Interestingly, an increasing number of studies have shown that skin senescence, a trigger of systemic ageing, is associated with organismal ageing and age‐related dysfunction.^4^, ^5^, ^6^, ^7^ Therefore, research on how to delay skin ageing or how to maintain skin homeostasis is of great significance.

Skin homeostasis is related to many factors such as oxidative stress, senescence‐associated secretory phenotype (SASP), apoptosis, autophagy and stem cell. Several studies have demonstrated that the level of oxidative stress and the balance of apoptosis and autophagy are crucial for maintaining skin homeostasis.^3^, ^8^, ^9^ This oxidative stress results in reduced collagen production, synthesis and activation of matrix metalloproteinases (MMPs) responsible for degrading connective tissue and secretion of SASP which ultimately promotes ageing of the skin.^3^, ^8^ Meanwhile, the balance of apoptosis and autophagy is perturbed by oxidative stress and inflammatory factors. In addition, stem cells underlie tissue homeostasis. Adult stem cells are essential to replace cells in tissues, but their capacity declines with age. Stem cell exhaustion or the accumulation of senescent cells has previously been reported to be implicated in ageing.^10^, ^11^, ^12^ Skin homeostasis is maintained through the presence of stem cells in epithelial tissues, which can replace those that are constantly lost during tissue turnover or following injury.^13^, ^14^ Recently, emphasis has been paid to the role of the hemidesmosome component collagen XVII (COL17A1) in stem cell competition and skin homeostasis.^15^, ^16^, ^17^ COL17A1 is not only a good marker for epidermal stem cells but also reflects the individual cellular potential and quality for self‐renewal. Hence, reducing the level of oxidative stress and the expression of SASP, regulating the balance of apoptosis and autophagy and enhancing the protein expression of COL17A1 may offer a new avenue for maintaining skin homeostasis and delaying skin ageing.

Traditional Chinese Medicine (TCM) employs the use of medicinal plants such as Ginger, Rutin, Astragalus mongholicus Bunge and Yang Yan Qing E Wan, which have unique advantages in alleviating skin senescence.^18^, ^19^, ^20^, ^21^, ^22^ Bazi Bushen is a Chinese patented drug approved by the National Medical Products Administration (No. B20020585) with the function of relieving fatigue and delaying ageing. Bazi Bushen consists of 16 botanical drugs, including *Cuscuate semen*, *Lycii fructus*, *Epimedii folium*, *Schisandrae chinensis fructus*, *Cnidii fructus*, *Rosae laevigatae fructus*, *Rubi fructus*, *Allii tuberosi semen*, *Radix morindae officinalis*, *Cistanches herba, Rehmanniae radix*, *Cyathulae radix*, *Ginseng radix et rhizoma*, *Cervi cornu pantotrichum*, *Hippocampus and Fuctus toosendan*. As described previously, the chemical profiles of Bazi Bushen were verified by ultra‐performance liquid chromatography (UPLC) analysis.^23^, ^24^ Bazi Bushen has been demonstrated to improve lipid metabolism, atherogenesis, ageing‐related hypogonadism and ageing‐associated cognitive impairments.^22^, ^23^, ^24^, ^25^ At the same time, these studies have also proven that Bazi Bushen could prevent skin senescence such as hair loss, skin atrophy and loose skin. However, the mechanism of Bazi Bushen on skin senescence remains largely unclear. In this study, we have preliminarily investigated the potential mechanism of Bazi Bushen on skin senescence in senescence‐accelerated mouse prone 6 (SAMP6). Our results demonstrated that Bazi Bushen could reduce the level of oxidative stress and the expression of SASP, regulate the balance of apoptosis and autophagy, and enhance the protein expression of COL17A1 in skin, thus maintaining skin homeostasis and delaying skin ageing.

## 2 METHODS AND MATERIALS

### 2.1 Chemicals and reagents

Bazi Bushen capsules were provided by Shijiazhuang Yiling Pharmaceutical Co., Ltd (Lot: A2102001, Shijiazhuang, China), according to the standard procedure approved by the National Medical Products Administration (No. B20020585). As described previously, the preparation of Bazi Bushen has been described in sufficient detail.^23^, ^24^ Tris‐Tricine‐SDS‐PAGE (No. P1200), BCA Protein Assay Kit (No. PC0020), SDS‐PAGE loading buffer, 5* (with DTT, No. P1040) and RAPI buffer (high, No. R0010) were purchased from Beijing Solarbio Science & Technology Co., Ltd. Commercial kits used for the determination of Hydroxyproline (Hyp, No. A030‐2‐1), Superoxide Dismutase (SOD, No. A001‐3‐2), Malondialdehyde (MDA, No. A005‐1‐2), Glutathione Peroxidase (GSH‐PX, No. A003‐1‐2) and Total antioxidant capacity (T‐AOC, No. A015‐2‐1) were purchased from Nanjing Jiancheng Bioengineering Institute. Mouse Interleukin‐6 (IL‐6, No. MM‐0163M1), Mouse Interleukin‐1β (IL‐1β, No. MM‐0040M1) and Mouse Tumour necrosis factor‐α (TNF‐α, No. MM‐0132M1) ELISA kits were purchased from Meimian Science Inc. Mouse Matrix metalloproteinase 2 (MMP2, No. JYM0019Mo), Mouse Matrix metalloproteinase 9 (MMP9, No. JYM0737Mo) and Mouse Vascular endothelial growth factor (VEGF, No. JYM0258Mo) ELISA kits were purchased from Jiyinmei Biological Technology Co., Ltd. Rabbit integrin subunit alpha 6 (ITGA6) Monoclonal Antibody BM4348 and Rabbit hemidesmosome component collagen XVII (COL17A1) Monoclonal Antibody BM4360 were purchased from Boster Biological Technology Co., Ltd. (Wuhan, China). Rabbit Bax Monoclonal Antibody ab32503, Rabbit Bcl‐2 polyclonal Antibody ab59348, Rabbit Cleaved caspase‐3 polyclonal Antibody ab13847 and Rabbit GAPDH monoclonal Antibody ab181602 were purchased from Abcam Co., Ltd. Rabbit LC3A/B monoclonal Antibody #12741, Rabbit SQSTM1/p62 monoclonal Antibody #23214 and Rabbit Beclin‐1 Antibody #3738 were purchased from Cell Signaling Technology. Rabbit P16‐INK4A polyclonal Antibody 10883‐1‐AP, Rabbit P21 polyclonal Antibody 10355‐1‐AP and Rabbit P53 polyclonal Antibody 10442‐1‐AP were purchased from Proteintech Group, Inc. Rabbit NANOG polyclonal Antibody A3232, Rabbit SOX2 polyclonal Antibody A0561 and Rabbit OCT4 polyclonal Antibody A7920 were purchased from ABclonal Technology Co., Ltd. HRP‐conjugated secondary antibody Goat Anti‐Rabbit IgG H&L ab205718, Goat Anti‐Rabbit IgG H&L (Alexa Fluor® 594) ab150080 and Goat Anti‐Rabbit IgG H&L (Alexa Fluor® 488) ab150077 were purchased from Abcam Co., Ltd.

### 2.2 Animals and treatments

Twenty male SAMP6 mice (25‐35 g) aged 9‐10 weeks were purchased from the Department of Laboratory Animal Science of Peking University (SCXK (JING) 2016–0010). The mice were acclimated in a 24.5 ± 0.5°C condition under a 12‐h light–dark cycle with water and food ad libitum. The experiments were approved by the Ethics Committee for the Welfare of Experimental Animals of Tianjin University of Traditional Chinese Medicine under the approval number TCM‐LAEC2021218. After 7 days of acclimation, the mice were randomly assigned to two groups (*n* = 10): SAMP6 and BZBS. The dosage of BZBS (2.8 g/kg/day) was based on the team's previous research data and literature reports.^22^, ^23^, ^24^, ^25^ The mice in the BZBS group were orally administrated with 2.8 g/kg/day of Bazi Bushen for 9 weeks. Body weights of mice were recorded, and the skin appearance was observed during the experiments.

### 2.3 Histological analysis of skin tissue

Skin tissue was fixed in 4% paraformaldehyde and embedded in paraffin, and 5 μm sections were stained with haematoxylin and eosin (H&E) and Masson's trichrome as described previously.^26^ The skin sample was examined using Nikon ELWD 0.3T1‐SNCP Microscope (Nikon, Japan). The dermal thickness was measured with Image J analyzer software.

### 2.4 Measurement of hydroxyproline (Hyp) content

According to the manufacturer's instruction,^27^ Hyp content in skin tissue was measured using commercial assay kits (Nanjing JianCheng Bioengineering Institute, Nanjing, China).

### 2.5 Measurement of oxidative damage sign

According to the manufacturer's instructions,^28^ SOD, MDA, GSH‐PX and T‐AOC in skin tissues were measured using commercial assay kits (Nanjing JianCheng Bioengineering Institute).

### 2.6 Enzyme‐linked immunosorbent assay (ELISA)

According to the manufacturer's instructions,^26^ IL‐6, IL‐1β, TNF‐α, MMP2, MMP9 and VEGF in skin tissues were measured using commercial ELISA kits. The microplate reader (Thermo, USA) was used to determine the optical density (OD) value at 450 nm.

### 2.7 Ageing‐related β‐galactosidase (SA‐β‐gal) staining

Skin tissue was fixed in 4% paraformaldehyde and embedded in paraffin, and 5 μm sections were incubated for 24 h, as reported previously.^29^ According to the manufacturer's instructions, the SA‐β‐gal staining kit (No. RG0039, Beyotime, China) was used for staining.

### 2.8 Immunofluorescence staining

Immunofluorescence was performed as described previously.^16^ Skin tissue was fixed in 4% paraformaldehyde and embedded in paraffin, and 5 μm sections were treated with xylene for deparaffinization. The sections were treated with 3% hydrogen peroxide to prevent endogenous peroxidase activity. After blocking with 10% goat serum for 1  h, the slides were incubated with ITGA6 and COL17A1 antibodies overnight. The sections were washed with PBS. Goat Anti‐Rabbit IgG H&L (Alexa Fluor® 594) and Goat Anti‐Rabbit IgG H&L (Alexa Fluor® 488) were used as the secondary antibodies, followed by the addition of 4′,6‐diamidine‐2′‐phenylindole dihydrochloride (DAPI, No. C0065, Solarbio, China). Coverslips were mounted on glass slides with a fluorescent mounting medium. All images were obtained using the IX2‐UCB confocal microscope system (Olympus, Japan).

### 2.9 Western blot analysis

Western blot analysis of the skin samples was conducted as described previously.^16^ The skin samples were put into an ice‐cold RIPA buffer solution for homogenate preparation. After centrifugation, we used a BCA kit to measure the protein concentration of the lysates. After denaturation, protein samples were separated using electrophoresis on SDS‐PAGE gels for about 90 min and then transferred onto PVDF membranes. Protein bands were blocked in QuickBlock Blocking Buffer for western blot (No. P0252, Beyotime, China) under gentle rocking for 15 min at RT and incubated with 1:1000 dilution of primary antibodies (ITGA6, GAPDH, Bax, Bcl‐2, Cleaved caspase‐3, LC3, p62, Beclin1, p16, p21, p53, OCT4, SOX2, NANOG and COL17A1) overnight at 4°C. Then, membranes were washed three times using TBST and probed with 1:10,000 dilution of HRP‐conjugated secondary antibody for 2 h at RT. Immunoblots were visualized using ECL reagents (No. 180–501, Biosharp, China), and band densities were quantified using Image J analyzer software. GAPDH was used as a normalization control. Each experiment was repeated at least three times.

### 2.10 Statistical analysis

PRISM8 software (Graph Pad Software) was used to assess statistical significance. To determine the significance between the two groups, comparisons were performed using an unpaired two‐tailed Student *t*‐test or Mann–Whitney U‐test for parametric and nonparametric data, respectively. *p* < 0.05 was considered statistically significant. **p < 0.01, *p < 0.05.

## 3 RESULTS

### 3.1 Bazi Bushen improved body weight and skin structure in SAMP6 mice

**FIGURE 1:**
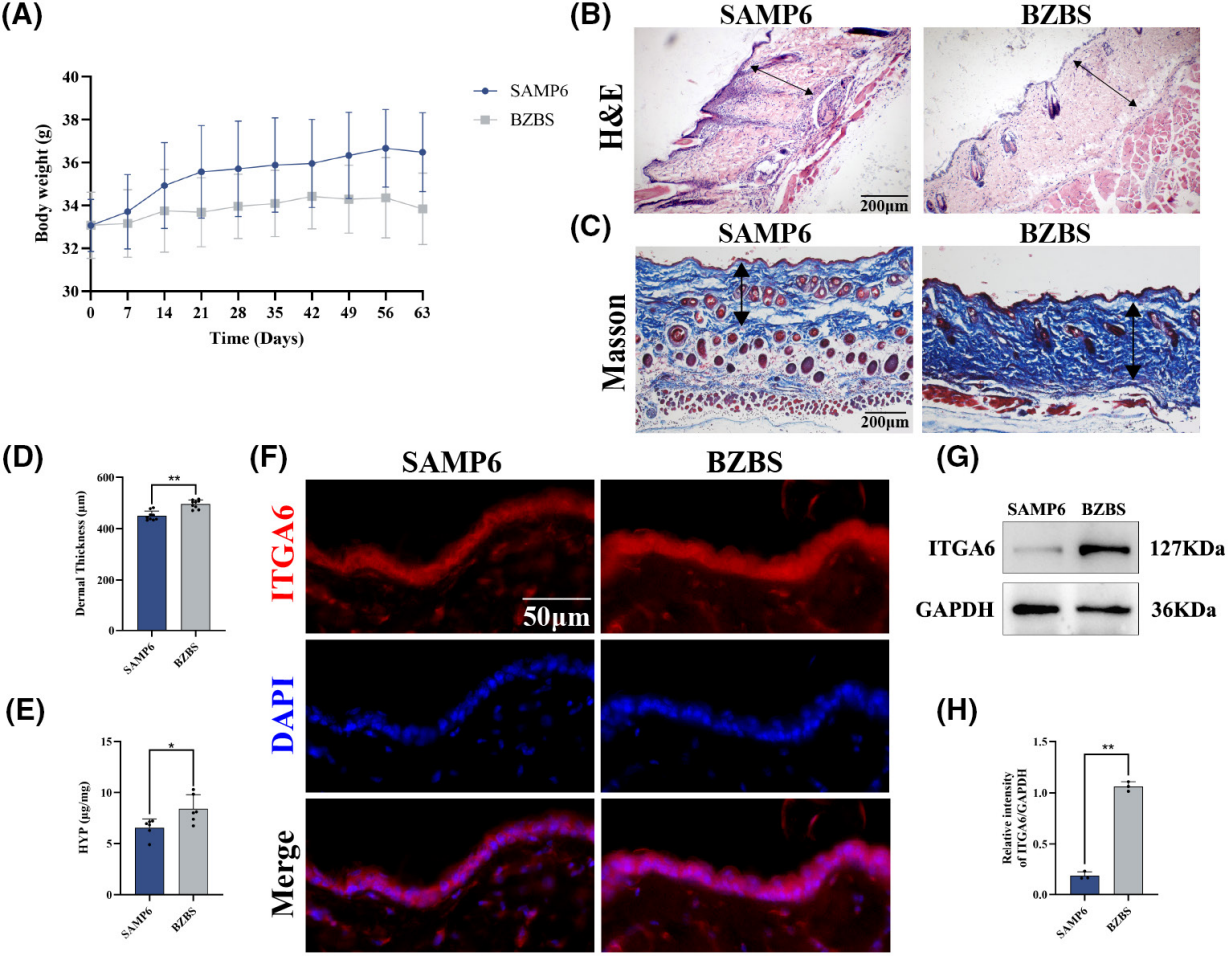
Effects of Bazi Bushen on body weight and skin structure in SAMP6 mice. (A) Body weight. (B) Representative haematoxylin and eosin images of skin structure. (C) Representative Masson images of skin structure. (D) The dermis thickness of skin tissues. (E) The contents of skin Hyp. (F) Representative immunofluorescence images of ITGA6+ cells in skin tissues. (G) Western blot analysis of ITGA6 and GAPDH in skin tissues. (H) Quantification of band intensities of ITGA6 relative to GAPDH in skin tissues. Data are expressed as mean ± SD. **p < 0.01 and *p < 0.05 versus the SAMP6 group (*n* = 4–6).

SAMP6 mice used to be a model of senile osteoporosis and exhibited obesity and loss of skin glossiness.^30^, ^31^, ^32^ During the experiments, body weight of mice was recorded and the skin appearance was observed. As shown in Figure 1A, the body weight decreased in the BZBS group compared with the SAMP6 group. We assumed that treatment with Bazi Bushen may improve the obesity of SAMP6 mice. Interestingly, compared with the BZBS group, the mice in the SAMP6 group exhibited hair loss and loose skin. To further explore the changes in the skin, we observed the skin structure by HE and Masson staining. The results revealed that dermis thickness of skin reduced, collagen fibres disordered and the amount of collagen decreased in the SAMP6 group compared with the BZBS group (Figure 1B–D). Furthermore, the skin Hyp content in the SAMP6 group was lower than that in the BZBS group (Figure 1E). To investigate the effects of Bazi Bushen on the hemidesmosome component, we measured the expression of ITGA6 by immunofluorescence staining and western blotting. The results revealed that the protein expression of ITGA6 increased in the BZBS group compared with the SAMP6 group (Figure 1F–H). These findings suggested that BZBS could improve body weight and skin structure in SAMP6 mice, and could rescue it from skin senescence.

### 3.2 Bazi Bushen delayed skin senescence in SAMP6 mice

**FIGURE 2:**
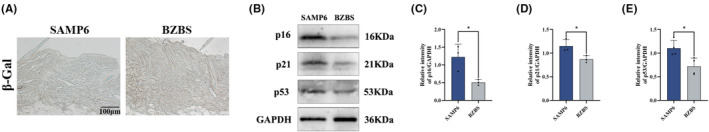
Effects of Bazi Bushen on skin senescence in SAMP6 mice. (A) Representative SA‐β‐gal images of skin structure. (B) Western blot analysis of p16, p21, p53 and GAPDH in skin tissues. (C) Quantification of band intensities of p16 relative to GAPDH in skin tissues. (D) Quantification of band intensities of p21 relative to GAPDH in skin tissues. (E) Quantification of band intensities of p53 relative to GAPDH in skin tissues. Data are expressed as mean ± SD. **p < 0.01 and *p < 0.05 versus the SAMP6 group (*n* = 3).

To further explore skin senescence visually, we observed the skin structure by SA‐β‐gal staining. We observed a decrease in SA‐β‐gal activity after Bazi Bushen treatment (Figure 2A). At the same time, we measured the expression of senescence markers p16, p21 and p53 using western blotting. In the BZBS group compared with the SAMP6 group, we observed a decrease in the levels of senescence markers p16, p21 and p53 (Figure 2B–E). These findings suggested that Bazi Bushen may delay skin senescence by preventing the senescence of internal cells and improving cell proliferation.

### 3.3 Bazi Bushen alleviated skin oxidative stress in SAMP6 mice

**FIGURE 3:**
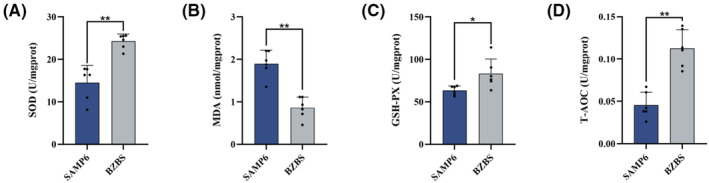
Effects of Bazi Bushen on skin oxidative stress in SAMP6 mice. (A) The contents of SOD in skin tissues. (B) The contents of MDA in skin tissues. (C) The contents of GSH‐PX in skin tissues. (D) The contents of T‐AOC in skin tissues. Data are expressed as mean ± SD. **p < 0.01 and *p < 0.05 versus the SAMP6 group (*n* = 6).

Oxidative stress regulates skin homeostasis and mediates the occurrence of skin senescence.^8^ Therefore, we examined the effect of Bazi Bushen on redox status in skin tissue of SAMP6 mice. As shown in Figure 3, compared with the SAMP6 group, Bazi Bushen treatment enhanced the activities of SOD, GSH‐PX and T‐AOC in skin tissue. Simultaneously, the skin tissue levels of MDA were significantly lower than those in the SAMP6 group. These findings indicated that Bazi Bushen could increase antioxidant capacity, which alleviated skin senescence.

### 3.4 Bazi Bushen decreased the levels of inflammatory cytokines in skin tissue of SAMP6 mice

**FIGURE 4:**
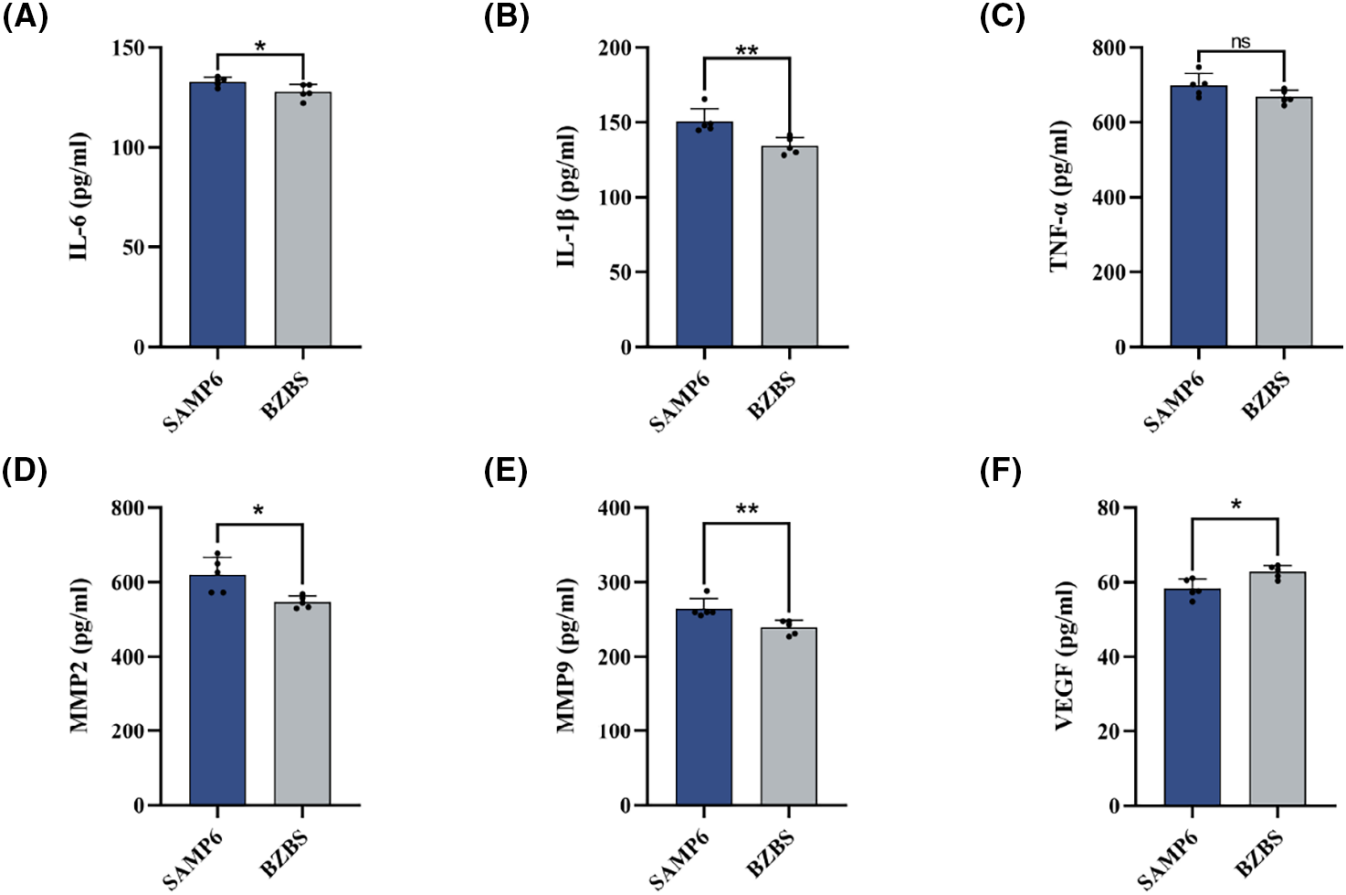
Effects of Bazi Bushen on skin SASP factors in SAMP6 mice. (A) The contents of IL‐6 in skin tissues. (B) The contents of IL‐1β in skin tissues. (C) The contents of TNF‐α in skin tissues. (D) The contents of MMP2 in skin tissues. (E) The contents of MMP9 in skin tissues. (F) The contents of VEGF in skin tissues. Data are expressed as mean ± SD. **p < 0.01 and *p < 0.05 versus the SAMP6 group (*n* = 5).

In addition, oxidative stress drives inflammageing.^3^ The release of inflammatory factors exacerbates oxidative damage. Both of them are essential for skin homeostasis. To confirm Bazi Bushen's effect on inflammation, we measured the levels of pro‐inflammatory cytokines in skin tissue. The results shown in Figure 4A–C compared the BZBS group with the SAMP6 group; we observed a decrease in the levels of pro‐inflammatory cytokines IL‐6, IL‐1β and TNF‐α. These findings indicated that Bazi Bushen could decrease the release of inflammatory factors, which may alleviate skin oxidative stress.

### 3.5 Bazi Bushen improved the levels of MMPs and VEGF in skin tissue of SAMP6 mice

The release of inflammatory factors increases the expression of MMPs, degrades collagen, leads to the loss of collagen and aggravates skin senescence.^8^ In ageing skin tissue, the expression of MMPs and the degradation of collagen degradation increased. At the same time, the decrease in micro‐vessels can lead to insufficient skin nutrient supply and slow down skin metabolism, thus accelerating skin senescence. To confirm Bazi Bushen's effect on skin MMPs and microcirculation, we measured the levels of MMP2, MMP9 and VEGF in skin tissue. The results revealed that Bazi Bushen treatment decreased the level of MMP2 and MMP9 and increased the level of VEGF (Figure 4D–F). These findings indicate that Bazi Bushen could improve the expression of skin MMPs and microcirculation.

### 3.6 Bazi Bushen regulated the balance of apoptosis and autophagy in skin tissue of SAMP6 mice

**FIGURE 5:**
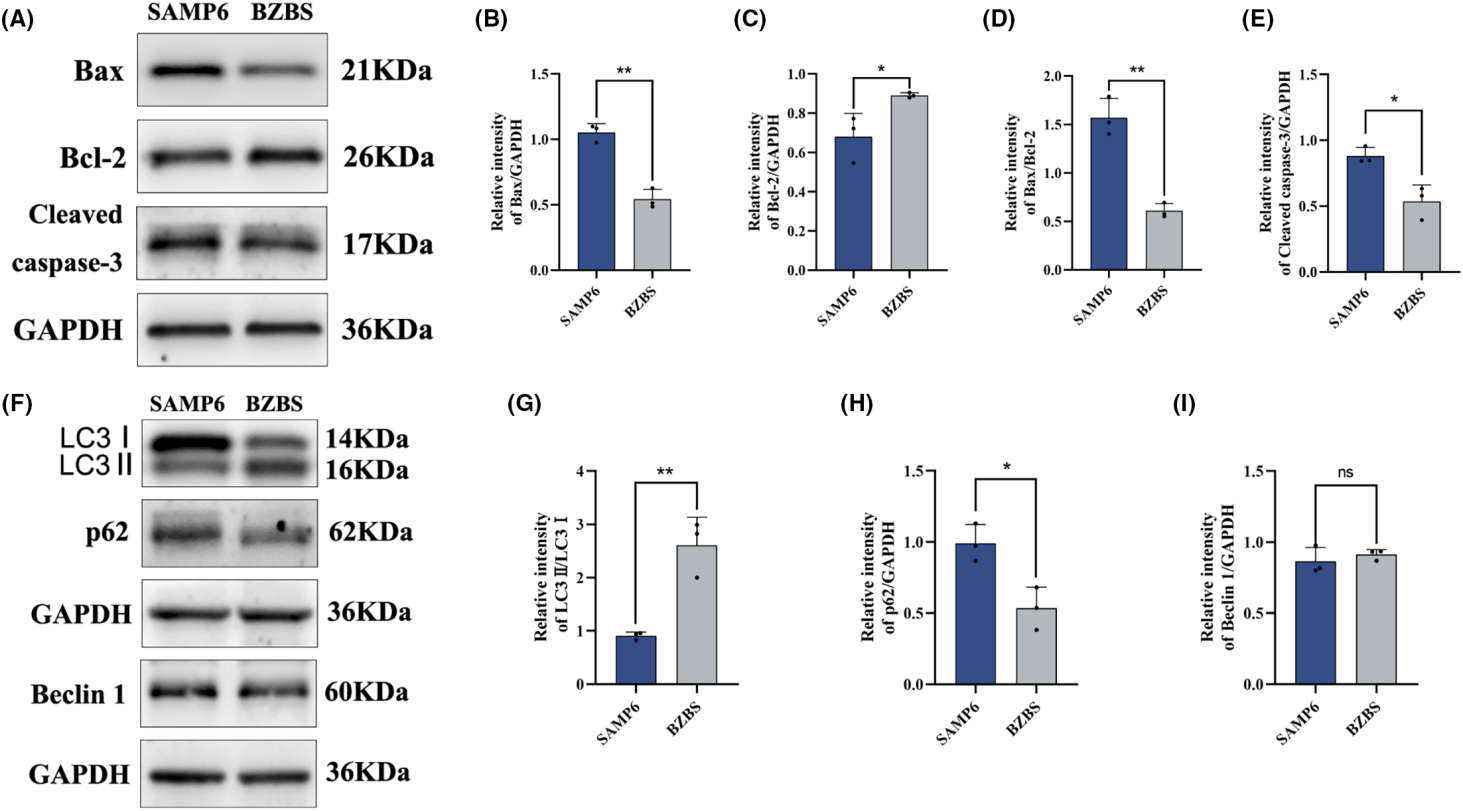
Effects of Bazi Bushen on skin apoptosis and autophagy in SAMP6 mice. (A) Western blot analysis of Bax, Bcl‐2, Cleaved caspase‐3 and GAPDH in skin tissues. (B) Quantification of band intensities of Bax relative to GAPDH in skin tissues. (C) Quantification of band intensities of Bcl‐2 relative to GAPDH in skin tissues. (D) Quantification of band intensities of Bax relative to Bcl‐2 in skin tissues. (E) Quantification of band intensities of Cleaved caspase‐3 relative to GAPDH in skin tissues. (F) Western blot analysis of LC3 I, LC3 II, p62, Beclin 1 and GAPDH in skin tissues. (G) Quantification of band intensities of LC3 II relative to LC3 I in skin tissues. (H) Quantification of band intensities of p62 relative to GAPDH in skin tissues. (I) Quantification of band intensities of Beclin 1 relative to GAPDH in skin tissues. Data are expressed as mean ± SD. **p < 0.01 and *p < 0.05 versus the SAMP6 group (*n* = 3).

Under normal conditions, a balance exists between apoptosis and autophagy that maintains skin homeostasis.^9^, ^33^ This balance is perturbed by oxidative stress and inflammatory factors such as skin senescence. Inflammation often causes apoptosis. In ageing skin tissue, pro‐apoptotic proteins increase and anti‐apoptotic proteins decrease. As expected, the expressions of pro‐apoptotic proteins Bax and Cleaved caspase‐3 were significantly decreased and the expression of anti‐apoptotic protein Bcl‐2 was markedly increased in skin tissue of the BZBS group compared with the SAMP6 group (Figure 5A–E). Autophagy is a cellular mechanism for eliminating damaged organelles.^34^ To confirm Bazi Bushen's effect on cell autophagy, we measured the conversion of LC3 I to LC3 II and the level of the autophagy receptor SQSTM1 and the autophagy protein Beclin 1 in skin tissue by western blotting. After treatment with Bazi Bushen, the conversion of LC3 I to LC3 II and the expression of the autophagy protein Beclin1 increased and the expression of the autophagy receptor SQSTM1 decreased in the BZBS group compared with the SAMP6 group, which indicated that the level of autophagy increased (Figure 5F–I). These findings indicate that Bazi Bushen may inhibit cell apoptosis and promote cell autophagy to alleviate skin senescence in SAMP6 mice.

### 3.7 Bazi Bushen regulated epidermal stem cells in SAMP6 mice

**FIGURE 6:**
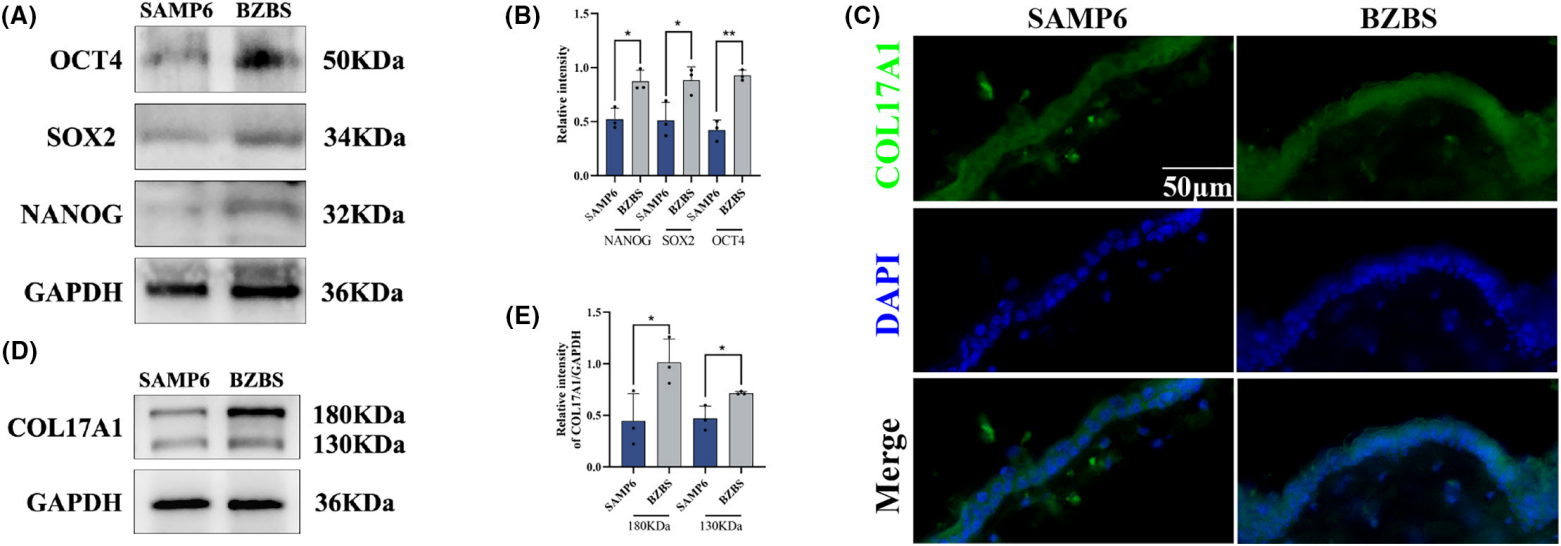
Effects of Bazi Bushen on epidermal stem cells in SAMP6 mice. (A) Western blot analysis of OCT4, SOX2, NANOG and GAPDH in skin tissues. (B) Quantification of band intensities of OCT4, SOX2 and NANOG relative to GAPDH in skin tissues. (C) Representative immunofluorescence images of COL17A1+ cells in skin tissues. (D) Western blot analysis of COL17A1 and GAPDH in skin tissues. (E) Quantification of band intensities of COL17A1 relative to GAPDH in skin tissues. Data are expressed as mean ± SD. **p < 0.01 and *p < 0.05 versus the SAMP6 group (*n* = 3).

Stem cells underlie tissue homeostasis.^16^ Adult stem cells are vital for replacing cells in tissues, but their capacity declines with age and stem cell exhaustion or the accumulation of senescent cells have previously been implicated in ageing.^10^, ^11^ To further explore the causes of structure and homeostasis changes in skin senescence, we measured the expression of stemness biomarkers NANOG, SOX2 and OCT4 in skin tissue by western blotting. In the BZBS group compared with the SAMP6 group, we observed an increase in the levels of stemness biomarkers (Figure 6A,B). To clarify whether Bazi Bushen can orchestrate skin homeostasis and senescence by intervening with stem cells, we measured the expression of the epidermal stem cells biomarker COL17A1 by immunofluorescence staining and western blotting. The results revealed that the protein expression of COL17A1 significantly increased in the BZBS group compared with the SAMP6 group (Figure 6C–E). These findings suggested that Bazi Bushen could orchestrate skin homeostasis and senescence by intervening with epidermal stem cells, which may therefore be beneficial for the reduction in ageing.

## 4 DISCUSSION

**FIGURE 7:**
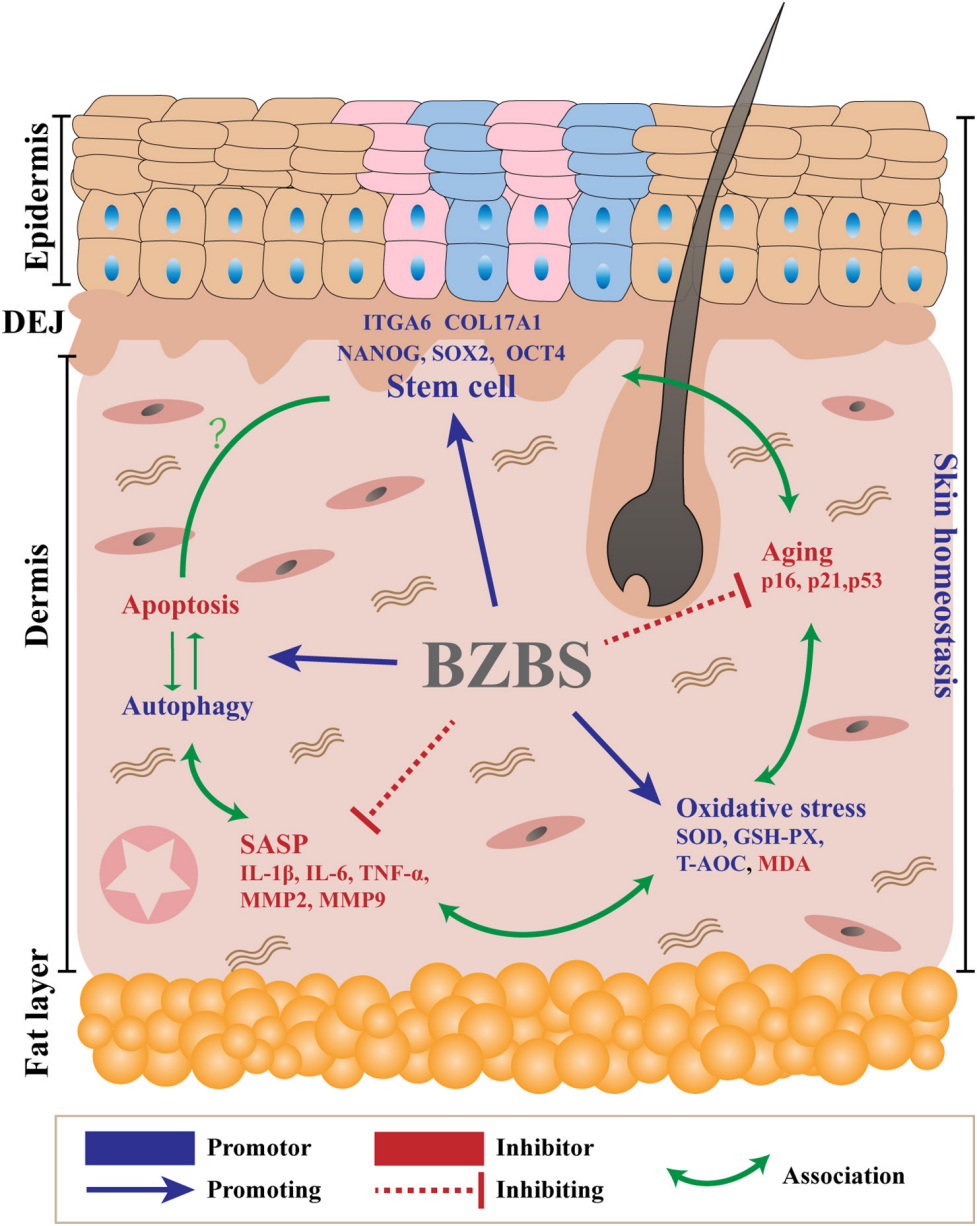
Mechanism by which Bazi Bushen alleviated skin senescence by orchestrating skin homeostasis.

In this study, we first demonstrated that Bazi Bushen alleviated skin senescence by orchestrating skin homeostasis in SAMP6 mice. As shown in Figure 7, Bazi Bushen delayed skin senescence by improving skin structure, reducing the level of oxidative stress and the expression of SASP, regulating the balance of apoptosis and autophagy and maintaining the protein expression of COL17A1 in SAMP6.

With the increasingly prominent problems of ageing population, environmental pollution and social pressure, the problems of skin ageing have attracted more attention. Skin ageing is associated with many factors, the most important of which is to orchestrate skin homeostasis. TCM exerts many health benefits, including ameliorative effects on skin ageing. By improving skin structure and orchestrating skin homeostasis, TCM can prevent or delay skin ageing. Bazi Bushen, a TCM formula, is used to relieve fatigue and delay ageing. As described previously, 14 compounds have been identified in Bazi Bushen, including neochlorogenic acid, chlorogenic acid, cryptochlorogenic acid, isoquercitrin, hyperin, verbascoside, epimedin A, icariin, baohuoside I, imperatorin, osthole, catalpol, deoxyschizandrin and γ‐Schizandrin.^22^, ^23^, ^24^, ^25^ These identified compounds may underlie the beneficial effects of Bazi Bushen to delay skin senescence.

The senescence‐accelerated mouse (SAM), consisting of senescence‐prone (SAMP) and senescence‐resistant (SAMR), has been developed and utilized worldwide for the study of human ageing and age‐associated diseases.^35^, ^36^ Compared with SAMR mice, SAMP mice showed signs of advanced senescence, such as loss of skin glossiness, hair loss and short life span.^30^ SAMP6 mice have used to be a model of senile osteoporosis and exhibited obesity and lower bone mass.^31^, ^32^ In the previous experiments, we first found that mice in the SAMP6 group exhibited lower bone mass compared with the SAMR1 group. Bazi Bushen treatment increased bone mineral density (BMD), bone mineral content (BMC), trabecular number (Tb.N), trabecular thickness (Tb.Th) and bone volume fraction (BV/TV). And in the process of experiments, we also found a remarkable phenomenon that mice in the SAMP6 group exhibited hair loss and loose skin compared with the BZBS group. This phenomenon was consistent with previous results.^23^, ^24^, ^25^ To preliminarily investigate the potential mechanism of Bazi Bushen on skin senescence, we tried to use SAMP6 mice as skin ageing model and conducted the following experiments.

By systematically investigating the pathophysiological changes in different tissues such as bone, skin, intestine, adipose and liver tissue after oral administration of Bazi Bushen, we confirmed that Bazi Bushen treatment alleviated various ageing‐related characteristics in multiple tissues, including inflammation, apoptosis, autophagy and fatty acid metabolism. Here, we only showed that Bazi Bushen exhibited a geroprotective effect on skin tissues in SAMP6 mice.

Skin structure, dermal thickness and the amount of collagen are commonly examined and considered symptoms of skin senescence. In addition, hemidesmosomes, connecting basal keratinocytes to the basement membrane, are crucial for an integral part of the skin.^16^ Our study demonstrated that Bazi Bushen improved skin structure, enhanced dermis thickness and increased the content of collagen and Hyp and the protein expression of ITGA6. Notably, the main compounds in BZBS, such as neochlorogenic acid,^37^ chlorogenic acid,^38^ cryptochlorogenic acid,^39^ isoquercitrin,^40^ hyperin,^41^ osthole^42^ and γ‐Schizandrin,^43^ could ameliorate collagen degradation, decreasing the level of MMPs, and delaying skin senescence. Altogether, our current results in combination with the previous findings support the beneficial role of Bazi Bushen in the prevention of skin senescence in ageing mice.

Antioxidants, such as SOD, GSH‐Px, CAT and GSH, play a critical role in defending against free radical damage to the human body.^8^ In the present study, Bazi Bushen treatment enhanced the activities of SOD, GSH‐PX and T‐AOC in skin tissue. Interestingly, various compounds and botanical drugs in Bazi Bushen have shown antioxidant activity.^24^, ^44^, ^45^, ^46^ Moreover, several literature studies have shown that TCM can reduce SASP secretion, promote cell autophagy and inhibit cell apoptosis.^47^, ^48^, ^49^ In our research, we examined senescence markers including SASP factors IL‐6, IL‐1β, TNF‐α, MMP2 and MMP9. The SASP was significantly decreased in treated Bazi Bushen compared with that in nontreated SAMP6 mice. And skin senescence can perturb the balance between apoptosis and autophagy, which contributes to promoting cell apoptosis and inhibiting cell autophagy. After treatment with Bazi Bushen, the situation has reversed.

Recently, an increasing number of studies have shown that stem cell competition orchestrates skin homeostasis and skin ageing.^16^ COL17A1 is a good marker for epidermal stem cells and reflects individual cellular potential and quality for self‐renewal. In the present study, we found that Bazi Bushen treatment enhanced the levels of stemness biomarkers NANOG, SOX2 and OCT4 in skin tissue. In addition, the expression of COL17A1 markedly increased. Altogether, these findings suggest that Bazi Bushen may alleviate skin senescence through the enhancement of stem cells and COL17A1 by orchestrating skin homeostasis in SAMP6 mice.

However, there are some limitations of the present study. We did not evaluate the effect of Bazi Bushen on the natural ageing mice. However, SAMP6 mice showed signs of advanced senescence, such as loss of skin glossiness, hair loss and short life span.^30^, ^31^ Meanwhile, treatment with Bazi Bushen can improve skin structure and delay skin senescence. The multidose studies will be needed and the dose–response curve in the experimental setup should be further clarified and detailed.^50^ And future investigations could evaluate the relationship between COL17A1 and the balance of apoptosis and autophagy.

## 5 CONCLUSION

In summary, based on our findings, Bazi Bushen can serve as a potential therapy for alleviating skin senescence. Its protective function on skin structure and homeostasis has been demonstrated, followed by its attenuation of oxidative stress and SASP, and regulation of the balance of apoptosis and autophagy in skin. The significance of COL17A1 in orchestrating skin homeostasis has also been revealed.

## AUTHOR CONTRIBUTIONS

**Zhe Xu:** Conceptualization (equal); data curation (equal); formal analysis (equal); methodology (equal); resources (equal); writing – original draft (equal). **Boyang Gong:** Investigation (equal). **Zhaodong Li:** Investigation (equal). **Ying Wang:** Investigation (equal). **Zeyu Zhao:** Resources (equal). **Lulu Xie:** Resources (equal). **Yanfei Peng:** Visualization (equal); writing – review and editing (equal). **Shuwu Zhao:** Visualization (equal); writing – review and editing (equal). **Huifang Zhou:** Conceptualization (equal); formal analysis (equal); methodology (equal); project administration (equal); supervision (equal). **Yuhong Bian:** Conceptualization (equal); formal analysis (equal); project administration (equal); supervision (equal); writing – review and editing (equal).

## FUNDING INFORMATION

This study was funded by the National Key R&D Program of China (2018YFC1706500) and the Strategic Consulting Project of the Chinese Academy of Engineering‐Strategic Research on Anti‐aging Effects of Traditional Chinese Medicine (2022‐XY‐45).

## CONFLICT OF INTEREST STATEMENT

All authors declare no conflict of interest.

## Data Availability

Data can be accessed by emailing the corresponding authors.

## References

[1] Partridge L , Deelen J , Slagboom PE . Facing up to the global challenges of ageing. Nature. 2018;561(7721):45‐56. doi:10.1038/s41586-018-0457-8 30185958

[2] Kubben N , Misteli T . Shared molecular and cellular mechanisms of premature ageing and ageing‐associated diseases. Nat Rev Mol Cell Biol. 2017;18(10):595‐609. doi:10.1038/nrm.2017.68 28792007 PMC6290461

[3] Gu Y , Han J , Jiang C , Zhang Y . Biomarkers, oxidative stress and autophagy in skin aging. Ageing Res Rev. 2020;59:101036. doi:10.1016/j.arr.2020.101036 32105850

[4] Gunn DA , de Craen AJM , Dick JL , et al. Facial appearance reflects human familial longevity and cardiovascular disease risk in healthy individuals. J Gerontol A Biol Sci Med Sci. 2013;68(2):145‐152. doi:10.1093/gerona/gls154 22879455

[5] Gunn DA , Larsen LA , Lall JS , Rexbye H , Christensen K . Mortality is written on the face. J Gerontol A Biol Sci Med Sci. 2016;71(1):72‐77. doi:10.1093/gerona/glv090 26265730 PMC4881821

[6] Waaijer MEC , Goldeck D , Gunn DA , et al. Are skin senescence and immunosenescence linked within individuals? Aging Cell. 2019;18(4):e12956. doi:10.1111/acel.12956 31062498 PMC6612632

[7] Franco AC , Aveleira C , Cavadas C . Skin senescence: mechanisms and impact on whole‐body aging. Trends Mol Med. 2022;28(2):97‐109. doi:10.1016/j.molmed.2021.12.003 35012887

[8] Kammeyer A , Luiten RM . Oxidation events and skin aging. Ageing Res Rev. 2015;21:16‐29. doi:10.1016/j.arr.2015.01.001 25653189

[9] Fernández ÁF , Sebti S , Wei Y , et al. Disruption of the beclin 1‐BCL2 autophagy regulatory complex promotes longevity in mice. Nature. 2018;558(7708):136‐140. doi:10.1038/s41586-018-0162-7 29849149 PMC5992097

[10] van Deursen JM . The role of senescent cells in ageing. Nature. 2014;509(7501):439‐446. doi:10.1038/nature13193 24848057 PMC4214092

[11] Goodell MA , Rando TA . Stem cells and healthy aging. Science. 2015;350(6265):1199‐1204. doi:10.1126/science.aab3388 26785478

[12] Childs BG , Gluscevic M , Baker DJ , et al. Senescent cells: an emerging target for diseases of ageing. Nat Rev Drug Discov. 2017;16(10):718‐735. doi:10.1038/nrd.2017.116 28729727 PMC5942225

[13] Blanpain C , Horsley V , Fuchs E . Epithelial stem cells: turning over new leaves. Cell. 2007;128(3):445‐458. doi:10.1016/j.cell.2007.01.014 17289566 PMC2408375

[14] Blanpain C , Fuchs E . Epidermal homeostasis: a balancing act of stem cells in the skin. Nat Rev Mol Cell Biol. 2009;10(3):207‐217. doi:10.1038/nrm2636 19209183 PMC2760218

[15] Matsumura H , Mohri Y , Binh NT , et al. Hair follicle aging is driven by transepidermal elimination of stem cells via COL17A1 proteolysis. Science. 2016;351(6273):aad4395. doi:10.1126/science.aad4395 26912707

[16] Liu N , Matsumura H , Kato T , et al. Stem cell competition orchestrates skin homeostasis and ageing. Nature. 2019;568(7752):344‐350. doi:10.1038/s41586-019-1085-7 30944469

[17] Nanba D , Toki F , Asakawa K , et al. EGFR‐mediated epidermal stem cell motility drives skin regeneration through COL17A1 proteolysis. J Cell Biol. 2021;220(11):e202012073. doi:10.1083/jcb.202012073 34550317 PMC8563287

[18] Chen F , Tang Y , Sun Y , Veeraraghavan VP , Mohan SK , Cui C . 6‐shogaol, a active constiuents of ginger prevents UVB radiation mediated inflammation and oxidative stress through modulating NrF2 signaling in human epidermal keratinocytes (HaCaT cells). J Photochem Photobiol B. 2019;197:111518. doi:10.1016/j.jphotobiol.2019.111518 31202076

[19] Choi SJ , Lee SN , Kim K , et al. Biological effects of rutin on skin aging. Int J Mol Med. 2016;38(1):357‐363. doi:10.3892/ijmm.2016.2604 27220601

[20] Cheng WJ , Chiang CC , Lin CY , et al. Astragalus mongholicus Bunge water extract exhibits anti‐inflammatory effects in human neutrophils and alleviates Imiquimod‐induced psoriasis‐like skin inflammation in mice. Front Pharmacol. 2021;12:762829. doi:10.3389/fphar.2021.762829 34955833 PMC8707293

[21] Tian LM , Peng Y , Ke D , et al. The effect of Yang Yan Qing E wan on senescent phenotypes and the expression of β‐catenin and p16INK4a in human skin fibroblasts. J Tissue Viability. 2020;29(4):354‐358. doi:10.1016/j.jtv.2020.06.001 32768331

[22] Li L , Zhang H , Chen B , et al. BaZiBuShen alleviates cognitive deficits and regulates Sirt6/NRF2/HO‐1 and Sirt6/P53‐PGC‐1α‐TERT signaling pathways in aging mice. J Ethnopharmacol. 2022;282:114653. doi:10.1016/j.jep.2021.114653 34547420

[23] Huang D , Wang X , Zhu Y , et al. Bazi Bushen capsule alleviates post‐menopausal atherosclerosis via GPER1‐dependent anti‐inflammatory and anti‐apoptotic effects. Front Pharmacol. 2021;12:658998. doi:10.3389/fphar.2021.658998 34248622 PMC8267998

[24] Li L , Chen B , An T , et al. BaZiBuShen alleviates altered testicular morphology and spermatogenesis and modulates Sirt6/P53 and Sirt6/NF‐κB pathways in aging mice induced by D‐galactose and NaNO2. J Ethnopharmacol. 2021;271:113810. doi:10.1016/j.jep.2021.113810 33508368

[25] Huang D , Hu H , Chang L , et al. Chinese medicine Bazi Bushen capsule improves lipid metabolism in ovariectomized female ApoE−/− mice. Ann Palliat Med. 2020;9(3):1073‐1083. doi:10.21037/apm-20-906 32434357

[26] Fan Y , Dong W , Wang Y , et al. Glycyrrhetinic acid regulates impaired macrophage autophagic flux in the treatment of non‐alcoholic fatty liver disease. Front Immunol. 2022;13:13. doi:10.3389/fimmu.2022.959495 PMC9365971 35967372

[27] Zhou M , Zhao X , Liao L , et al. Forsythiaside A regulates activation of hepatic stellate cells by inhibiting NOX4‐dependent ROS. Oxid Med Cell Longev. 2022;2022:9938392. doi:10.1155/2022/9938392 35035671 PMC8754607

[28] Xu L , Yu Y , Sang R , Li J , Ge B , Zhang X . Protective effects of taraxasterol against ethanol‐induced liver injury by regulating CYP2E1/Nrf2/HO‐1 and NF‐κB signaling pathways in mice. Oxid Med Cell Longev. 2018;2018:8284107. doi:10.1155/2018/8284107 30344887 PMC6174809

[29] Wang Z , Wang L , Jiang R , et al. Ginsenoside Rg1 prevents bone marrow mesenchymal stem cell senescence via NRF2 and PI3K/Akt signaling. Free Radical Bio Med. 2021;174:182‐194. doi:10.1016/j.freeradbiomed.2021.08.007 34364981

[30] Suzuki T , Aoki K , Shimokobe K , et al. Age‐related morphological and functional changes in the small intestine of senescence‐accelerated mouse. Exp Gerontol. 2022;163:111795. doi:10.1016/j.exger.2022.111795 35378239

[31] Niimi K , Takahashi E , Itakura C . Adiposity‐related biochemical phenotype in senescence‐accelerated mouse prone 6 (SAMP6). Comp Med. 2009;59(5):431‐436. 19887026 PMC2771603

[32] Azuma K , Zhou Q , Kubo KY . Morphological and molecular characterization of the senile osteoporosis in senescence‐accelerated mouse prone 6 (SAMP6). Med Mol Morphol. 2018;51(3):139‐146. doi:10.1007/s00795-018-0188-9 29619545

[33] Liu J , Liu W , Lu Y , et al. Piperlongumine restores the balance of autophagy and apoptosis by increasing BCL2 phosphorylation in rotenone‐induced Parkinson disease models. Autophagy. 2018;14(5):845‐861. doi:10.1080/15548627.2017.1390636 29433359 PMC6070010

[34] Klionsky DJ , Abdel‐Aziz AK , Abdelfatah S , et al. Guidelines for the use and interpretation of assays for monitoring autophagy (4th edition)^1^ . Autophagy. 2021;17(1):1‐382. doi:10.1080/15548627.2020.1797280 33634751 PMC7996087

[35] Takeda T , Hosokawa M , Higuchi K . Senescence‐accelerated mouse (SAM): a novel murine model of senescence. Exp Gerontol. 1997;32(1–2):105‐109. doi:10.1016/s0531-5565(96)00036-8 9088907

[36] Takeda T . Senescence‐accelerated mouse (SAM): a biogerontological resource in aging research. Neurobiol Aging. 1999;20(2):105‐110. doi:10.1016/s0197-4580(99)00008-1 10537019

[37] Ahn HS , Kim HJ , Na C , Jang DS , Shin YK , Lee SH . The protective effect of *Adenocaulon himalaicum* Edgew. And its Bioactive Compound Neochlorogenic Acid against UVB‐induced skin damage in human dermal fibroblasts and epidermal keratinocytes. Plants (Basel). 2021;10(8):1669. doi:10.3390/plants10081669 34451713 PMC8399472

[38] Her Y , Lee TK , Kim JD , et al. Topical application of *Aronia melanocarpa* extract rich in Chlorogenic acid and Rutin reduces UVB‐induced skin damage via attenuating collagen disruption in mice. Molecules. 2020;25(19):E4577. doi:10.3390/molecules25194577 PMC7582310 33036412

[39] Yi R , Zhang J , Sun P , Qian Y , Zhao X . Protective effects of kuding tea (Ilex kudingcha C. J. Tseng) polyphenols on UVB‐induced skin aging in SKH1 hairless mice. Molecules. 2019;24(6):E1016. doi:10.3390/molecules24061016 PMC6470819 30871261

[40] Lee EH , Park HJ , Kim HH , Jung HY , Kang IK , Cho YJ . Isolated isoquercitrin from green ball apple peel inhibits photoaging in CCD‐986Sk fibroblasts cells via modulation of the MMPs signaling. J Cosmet Dermatol‐US. 2021;20(9):2932‐2939. doi:10.1111/jocd.13903 33356000

[41] Mapoung S , Umsumarng S , Semmarath W , et al. Photoprotective effects of a Hyperoside‐enriched fraction prepared from *Houttuynia cordata* Thunb. On ultraviolet B‐induced skin aging in human fibroblasts through the MAPK signaling pathway. Plants (Basel). 2021;10(12):2628. doi:10.3390/plants10122628 34961096 PMC8708340

[42] Tsai YF , Chen CY , Lin IW , et al. Imperatorin alleviates psoriasiform dermatitis by blocking neutrophil respiratory burst, adhesion, and chemotaxis through selective phosphodiesterase 4 inhibition. Antioxid Redox Sign. 2021;35(11):885‐903. doi:10.1089/ars.2019.7835 33107318

[43] Chen D , Du Z , Lin Z , et al. The chemical compositions of Angelica pubescens oil and its prevention of UV‐B radiation‐induced cutaneous photoaging. Chem Biodivers. 2018;15(10):e1800235. doi:10.1002/cbdv.201800235 29996001

[44] Szeto YT , Wong SCY , Wong JWM , Kalle W , Pak SC . In vitro antioxidation activity and genoprotective effect of selected Chinese medicinal herbs. Am J Chin Med. 2011;39(4):827‐838. doi:10.1142/S0192415X11009238 21721160

[45] Han X , Shen S , Liu T , et al. Characterization and antioxidant activities of the polysaccharides from radix Cyathulae officinalis Kuan. Int J Biol Macromol. 2015;72:544‐552. doi:10.1016/j.ijbiomac.2014.09.007 25236610

[46] Wang W , Wang S , Liu J , et al. Sesquiterpenoids from the root of Panax Ginseng protect CCl4‐induced acute liver injury by anti‐inflammatory and anti‐oxidative capabilities in mice. Biomed Pharmacother. 2018;102:412‐419. doi:10.1016/j.biopha.2018.02.041 29573620

[47] Gao L , Yang WY , Qi H , Sun CJ , Qin XM , Du GH . Unveiling the anti‐senescence effects and senescence‐associated secretory phenotype (SASP) inhibitory mechanisms of Scutellaria baicalensis Georgi in low glucose‐induced astrocytes based on boolean network. Phytomedicine. 2022;99:153990. doi:10.1016/j.phymed.2022.153990 35202958

[48] Kang X , Chen L , Yang S , et al. Zuogui wan slowed senescence of bone marrow mesenchymal stem cells by suppressing Wnt/β‐catenin signaling. J Ethnopharmacol. 2022;294:115323. doi:10.1016/j.jep.2022.115323 35483559

[49] Li H , Yang T , Tian LM , Zhang PC . Effects of Qi‐Bao‐Mei‐ran‐Dan on proliferative activity and expressions of apoptosis‐related genes Bcl‐2, Bax and autophagy‐related protein LC3II in fibroblasts of aging skin. Asian J Surg. 2022;45(6):1319‐1321. doi:10.1016/j.asjsur.2022.01.043 35367098

[50] Heinrich M , Appendino G , Efferth T , et al. Best practice in research–overcoming common challenges in phytopharmacological research. J Ethnopharmacol. 2020;246:112230. doi:10.1016/j.jep.2019.112230 31526860

